# Euthyroid Sick Syndrome as an Index of Prognosis in Severe COVID-19 Disease

**DOI:** 10.3390/medicina61081372

**Published:** 2025-07-29

**Authors:** Lambros Athanassiou, Ifigenia Kostoglou-Athanassiou, Georgia Kaiafa, Sofia Nikolakopoulou, Alexandra Konstantinou, Olga Mascha, Charilaos Samaras, Christos Savopoulos, Yehuda Shoenfeld, Panagiotis Athanassiou

**Affiliations:** 1 COVID-19 Department, Asclepeion Hospital, Voula, 16673 Athens, Greece; lambros.ath@gmail.com (L.A.); sofnikol@yahoo.gr (S.N.); alexkonstantinou2@gmail.com (A.K.); xarisamar@gmail.com (C.S.); 2 Department of Endocrinology, Diabetes, Metabolism, Asclepeion Hospital, Voula, 16673 Athens, Greece; 3 First Propaedeutic Department of Internal Medicine, AHEPA University General Hospital, Aristotle University of Thessaloniki, 54636 Thessaloniki, Greecechrisavopoulos@gmail.com (C.S.); 4 Department of Biochemistry, Asclepeion Hospital, Voula, 55134 Athens, Greece; olmascha@gmail.com; 5 Medical School, Reichman University, Herzliya 4610101, Israel; yehuda.shoenfeld@sheba.health.gov.il; 6 Department of Rheumatology, St. Paul’s Hospital, 55134 Thessaloniki, Greece; pathanassiou@yahoo.gr

**Keywords:** SARS-CoV-2, COVID-19, euthyroid sick syndrome, non-thyroidal illness syndrome

## Abstract

*Background and Objectives*: Euthyroid sick syndrome, or non-thyroidal illness syndrome, has been observed in severely ill patients and has been found to be an index of prognosis. It has been detected in patients with severe infectious diseases, e.g., those with severe COVID-19 infection. Prognostic indicators of the outcome of severe COVID-19 disease are important for the prognosis of individual as well as groups of patients. The aim of this study was to identify euthyroid sick syndrome in patients admitted for severe COVID-19 disease and its relationship to disease severity and outcome. *Materials and Methods*: In a cohort of patients admitted to hospital for severe COVID-19 disease, thyroid function in patients requiring hospitalization was evaluated by measuring TSH, FreeT_3_ (FT_3_), and FreeT_4_ (FT_4_) levels. Patients were classified into four groups: a group with uncompromised respiratory function (pO2 > 70 mmHg, without need of oxygen supplementation) (disease severity 1); a group with mild respiratory insufficiency (pO2 50–60 mmHg, in need of oxygen supplementation with nasal cannula) (disease severity 2); a group with severe respiratory insufficiency (pO2 < 50 mmHg, in need of oxygen supplementation with high flow oxygen) (disease severity 3); and a group with severe respiratory insufficiency requiring intubation (pO2 < 60 mmHg on high flow oxygen supplementation) (disease severity 4). *Results*: In this cohort, euthyroid sick syndrome was diagnosed in 57.1% of the patients. The presence of euthyroid sick syndrome was related to increased disease severity and adverse disease outcome, i.e., death. FT_3_ levels were inversely related to CRP levels. *Conclusions*: Euthyroid sick syndrome may be observed in severe COVID-19 disease and is related to increased disease severity and adverse outcomes. Measurement of thyroid hormones in patients hospitalized for severe COVID-19 infection may aid in the prognosis of the disease.

## 1. Introduction

Euthyroid sick syndrome or low T_3_ or non-thyroidal illness syndrome is a condition induced by severe illness in patients without thyroid or hypothalamic and pituitary disease [1,2]. It reflects an adaptation of the thyroid to severe disease [2]. The condition was first described in the 1970s, as it was observed that thyroid hormone levels dropped during starvation and illness. In a state of mild illness, T_3_ levels decreased, but as illness duration and severity increased, T_4_ levels decreased as well. This drop in thyroid hormone levels was observed in a variety of disease states. In contrast to hypothyroidism, decreased peripheral thyroid hormone levels were not followed by an increase in TSH levels. Following these early observations, various studies investigated alterations in thyroid hormone secretion observed during acute illness [3]. However, the exact pathophysiology of euthyroid sick syndrome is still under investigation.

Various mechanisms are involved in the pathophysiology of euthyroid sick syndrome. Modulation of deiodinase activity—in particular, upregulation of deiodinase type 1 (D1) and type 3 (D3)—leads to decreased production of FT_3_ and increased production of the inactive compound rT_3_ [2], respectively [4,5]. Modulation of hypothalamic pituitary secretory activity, i.e., decreased TRH secretion due to higher inhibitory signals or impaired pulsatile TSH, leads to decreased TSH levels. Leptin levels decrease as the illness progresses and lead to decreased TRH secretion and thereby decreased TSH levels [6]. Thyroid hormone binding to transport proteins is modulated and may lead to the profile of euthyroid sick syndrome [2]. Modulated T_3_ binding to its receptors may also contribute to its pathogenesis [2].

Euthyroid sick syndrome may be observed in various situations and disease states, including cases of starvation [7,8,9], patients in the intensive care unit [3], patients undergoing cardiac operations [10,11], premature infants [3,12], severely ill pediatric and adult patients requiring extracorporeal circulatory support [13], patients with hip fractures [14], patients with acute complications of diabetes mellitus such as diabetic ketoacidosis [15], patients with renal failure [16] as well as severely ill patients suffering from various conditions but not cared for in the intensive care unit [17]. In acute coronary artery disease events as well as in patients under acute care, severe euthyroid sick syndrome may be observed [18,19], and it is considered a prognostic indicator of disease outcome, i.e., survival or death [20].

The secretory activity of the hypothalamic pituitary unit is modulated by circulating inflammatory cytokines during acute illness, leading to the hormone profile of euthyroid sick syndrome. Modulation of deiodinase activity has been considered a major mechanism leading to decreased secretion of T_3_ and FT_3_ and augmented secretion of rT_3_. RT_3_ is a thyroid hormone product and is deprived of the major actions of thyroid hormones. Modulation of thyroid hormone metabolism during acute disease is also considered another mechanism involved in the pathophysiology of euthyroid sick syndrome. Modulation of the conjugation of circulating thyroid hormones with thyroid binding proteins and albumin is also a mechanism considered to contribute to its pathogenesis [21].

Euthyroid sick syndrome is mainly characterized by the presence of low FT_3_, which is not accompanied by a compensatory increase in TSH levels but by a decrease. As the underlying disease state deteriorates, low FT_4_ levels may be observed [22]. These alterations reflect the effect of severe illness on inflammatory mediator secretion which affects the neuroendocrine and endocrine axis [23].

Euthyroid sick syndrome is also observed in infectious diseases [21]. The condition has been previously described in pulmonary tuberculosis [24], in severe meningococcal infection [21], and in human immunodeficiency virus infection [25]. Chow et al. [24] studied a cohort of patients with pulmonary tuberculosis and estimated thyroid hormone secretion. They observed euthyroid sick syndrome in 63% of their cohort, and they found that a very low FT_3_ at presentation was related to subsequent mortality. Post et al. [26] studied endocrine function in active tuberculosis patients and found that 92% of the patients had euthyroid sick syndrome at presentation. Den Brinker et al. [21] studied euthyroid sick syndrome in pediatric patients with meningococcal sepsis. They found that all their pediatric patients with severe meningococcal infection showed alterations in thyroid hormone levels, indicating the diagnosis of non-thyroidal illness syndrome. Altered thyroid hormone levels were inversely related to disease duration and could be due to type 3 deiodinase (D3) induction. In addition, dopamine was found to suppress TSH levels, and T4 and IL-6 levels were predictive of mortality. In the context of COVID-19 infection, euthyroid sick syndrome has been reported [27] and serves as a marker of disease outcome. The relationship between euthyroid sick syndrome and outcomes of death has been described in the literature [20,28,29,30]. It was found to be a marker of adverse outcomes in elderly patients who had undergone surgery for emergency situations [28], in patients with cardiac insufficiency [29], in patients with coronary artery disease [20,30], and in patients with sepsis [31].

The aim of this study was to evaluate thyroid function and investigate euthyroid sick syndrome and its relationship with disease severity and prognosis in a group of patients hospitalized for severe COVID-19 infection.

## 2. Materials and Methods

In a group of 63 patients admitted to the COVID-19 Department of Asclepeion Hospital, Voula, Athens, Greece, over a period of 12 months, from April 2021 to the end of March 2022, for severe COVID-19 infection, TSH, FT_3_, FT_4_, and CRP levels were measured. Thyroid hormone measurement was performed upon admission to the COVID-19 Department. Patients underwent evaluation and treatment for severe COVID-19 disease, which was defined as disease requiring hospitalization in the COVID-19 Department. The patients were classified into four groups: a group with uncompromised respiratory function (pO2 > 70 mmHg, without need of oxygen supplementation) (disease severity 1); a group with mild respiratory insufficiency (pO2 50–60 mmHg, in need of oxygen supplementation with nasal cannula) (disease severity 2); a group with severe respiratory insufficiency (pO2 < 50 mmHg, in need of oxygen supplementation with high flow oxygen) (disease severity 3); and a group with severe respiratory insufficiency requiring intubation (pO2 < 60 mmHg on high flow oxygen supplementation, displaying tachypnea and tachycardia) (disease severity 4) (Table 1). Patients had either one, two, or three comorbidities, i.e., arterial hypertension, diabetes mellitus, and coronary artery disease. Patients who had a history of thyroid disease or were taking medications for the treatment of an underlying thyroid disease were excluded from the study.

TSH, Free T_3_ (FT_3_), and Free T_4_ (FT_4_) levels were measured by a chemiluminescence immunoassay, which is a laboratory method applied to detect analytes in biological fluids by the generation of light via a chemical reaction between specific antibodies and labeled molecules.

TSH levels were assayed in serum by the application of the ARCHITECT TSH immunoassay (Abbott Park IL), a chemiluminescent microparticle immunoassay. The analytical sensitivity of the immunoassay was <0.0025 μIU/mL, the precision was <10%, and the inter-assay coefficient of variation was <20%; normal values were 0.35–4.94 μIU/mL. The assay uses flexible protocols, known as Chemiflex. The assay is based on acridinium labeled conjugate and the detection of relative light units by an optical system.

FT_3_ levels were assayed by the ARCHITECT FT3 assay (Abbott Park IL). The analytical sensitivity of the immunoassay was <1.0 pg/mL, and the analytical specificity was <0.001%; normal values were 1.58–3.91 pg/mL. The assay is based on acridinium labeled conjugate which is added to the reaction mixture.

FT_4_ levels were assayed by the ARCHITECT FT4 assay (Abbott Park IL). The analytical sensitivity of the immunoassay was <0.4 ng/dL, and the precision was <10%; normal values were 0.70–1.48 ng/dL. The assay is based on the inverse relationship between the amount of FT_4_ in the sample and the light units emitted during the reaction and detected by an optical system.

CRP was estimated by particle-enhanced immunonephelometry (CardioPhase hsCRP, Siemens Helathcare Diagnostics, Camerley, UK), a method with a sensitivity and intra-assay and inter-assay coefficients of variation of 0.175 mg/L with a CV of 7.6% at 0.41 mg/L, 2.7% at 14 mg/L, and 2.0% at 14 mg/L, respectively. Normal values for CRP were <5 mg/L.

The subjects included in the study had no known history of thyroid disease and were not taking any medication for thyroid disease.

Euthyroid disease was diagnosed if patients without a known history of thyroid disease had low FT_3_ levels in the presence of normal or low TSH levels.

The study was approved by the ethical committee of Asclepeion Hospital, Voula, Athens, Greece (approval number 15335, 24 November 2020). All the patients involved in the study gave their informed consent.

The statistical evaluation of the data generated in the study was performed by SPSS (IBP SPSS Statistics v27). Data are shown as mean ± SD. For the statistical evaluation of qualitative data, a chi square test was performed. For the statistical evaluation of quantitative data, ANOVA and linear regression analysis were performed.

## 3. Results

### 3.1. Study Population

Patients underwent evaluation and treatment for severe COVID-19 infection (Table 1).

### 3.2. Identification of Euthyroid Sick Syndrome

Euthyroid sick syndrome, as assessed by low FT_3_ and normal or low TSH levels, was observed in 36 patients in this cohort (57.1%), while 27 patients did not have such evidence.

Euthyroid sick syndrome was related to adverse outcomes, i.e., non-survival or death, and to disease severity (*p* < 0.001, chi square test).

### 3.3. Laboratory Findings

**Figure 1:**
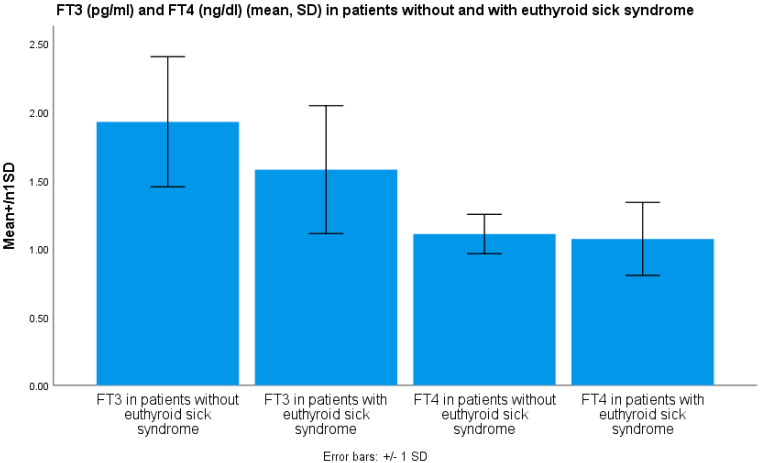
FT_3_ (pg/mL, mean ± SD) and FT_4_ (ng/dL, mean ± SD) levels in patients with severe COVID-19 disease without and with euthyroid sick syndrome.

FT_3_ levels in the group of patients diagnosed with euthyroid sick syndrome (1.59 ± 0.31 pg/mL) were lower compared to patients without evidence of euthyroid sick syndrome (2.26 ± 0.31 pg/mL) (mean ± SD) (*p* < 0.001, ANOVA) (shown in Figure 1). FT_3_ levels were lower in male and female patients with COVID-19 infection with euthyroid sick syndrome compared to those without euthyroid sick syndrome (*p* < 0.001). 

FT_4_ levels were lower in the group of patients with euthyroid sick syndrome (1.07 ± 0.19 ng/dL) as opposed to the patients without euthyroid sick syndrome (1.18 ± 0.18 ng/dL) (mean ± SD, *p* < 0.05 ANOVA) (Figure 1). FT_4_ levels were lower in male and female patients with COVID-19 infection with euthyroid sick syndrome as opposed to those without the syndrome (*p* < 0.05).

**Figure 2:**
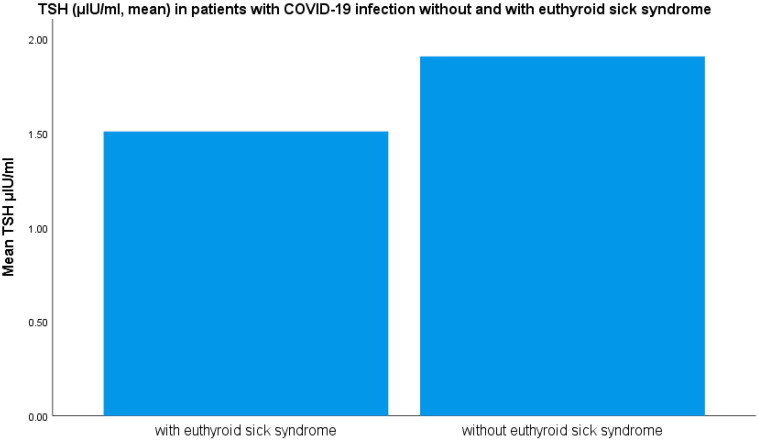
TSH levels (μIU/mL) (mean ± SD) in patients with severe COVID-19 infection with and without euthyroid sick syndrome.

TSH levels were lower in the group of patients with euthyroid sick syndrome (1.26 ± 1.68 μIU/mL) as opposed to the patients without euthyroid sick syndrome (1.64 ± 1.8 μIU/mL) (mean ± SD, *p* < 0.05 ANOVA) (Figure 2). TSH levels were lower in male and female patients with COVID-19 infection with euthyroid sick syndrome as opposed to those without the syndrome (*p* < 0.05).

**Figure 3:**
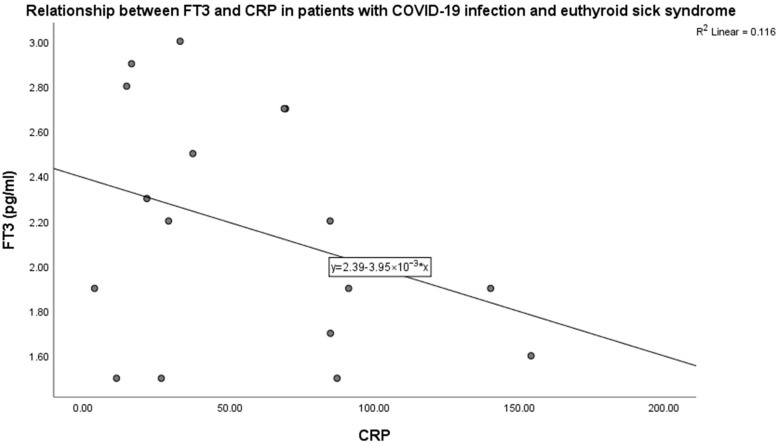
Relationship between FT_3_ and CRP levels in patients with severe SARS-CoV-2 infection.

FT_3_ levels were inversely related to CRP levels in patients with severe COVID-19 infection (*p* < 0.001, beta coefficient of variation —0.34, linear regression analysis) (Figure 3).

## 4. Discussion

Euthyroid sick syndrome or non-thyroidal illness syndrome represents the response of the organism to acute and severe illness [2,3]. In a state of acute illness, a patient may present with reduced levels of T_3_ and normal or decreased TSH levels. In contrast, plasma rT_3_ levels increase and plasma T_4_ levels are low or low–normal. These thyroid hormone alterations are observed in all age groups, ranging from neonatal age and childhood to adulthood and old age [12]. The severity of the underlying condition is correlated with the euthyroid sick syndrome phenotype. Although euthyroid sick syndrome was described many years ago, the pathophysiology of the condition remains incompletely understood. The pathophysiology of euthyroid sick syndrome is multifactorial. In acute disease, thyroid hormones are inactivated peripherally. These changes, which are observed in the circulation, may represent a beneficial adaptation to illness aiming to reduce energy consumption. Peripheral inactivation of thyroid hormones leads to activation of the innate immune response in order to enable survival [32]. In the case of more severe and prolonged illness, in addition to peripheral thyroid hormone changes, the thyroid hormone axis is centrally suppressed, leading to a more severe euthyroid hormone sick phenotype. In severe, critical, and prolonged disease, the presence of a centrally suppressed thyroid hormone axis leads to aggravation of the euthyroid sick syndrome phenotype [33]. Euthyroid sick syndrome is found in patients suffering from infectious diseases and has been observed in ill children with multisystem inflammatory syndrome [34] and severe disease course.

Euthyroid sick syndrome has been observed in patients with COVID-19 virus infection [27]. In a retrospective study performed at the Sheba Medical Center in Israel [35], it was found that, in a cohort of COVID-19 patients, those with FT_3_ levels in the lower tertile had significantly higher mortality, need for mechanical ventilation, and need for specialized acute care. In a clinical study performed in Changsha, China, non-thyroidal illness syndrome was found to be related to severe disease and inflammatory parameters in COVID-19 disease [27]. In an observational study performed in London, thyroid function was evaluated in patients with COVID-19 infection [36], showing decreased FT_4_ and TSH levels in comparison to baseline historical levels of the same patients, where available. FT_4_ and TSH levels increased in the survivors after recovery from the infection. In a retrospective study performed in Lodz, Poland, amongst patients hospitalized for COVID-19 infection, it was found that euthyroid sick syndrome was related to higher inflammatory indices, longer hospitalization, and the need for oxygen therapy or intubation, as compared to those without euthyroid sick syndrome [37]. In the aforementioned study, mortality was higher in those suffering from euthyroid sick syndrome and <50% of lung parenchymal involvement on computer tomography. In a prospective study of one year duration, performed in Florence, Italy, in patients hospitalized with mild COVID-19 infection, it was found that euthyroid sick syndrome, as depicted by decreased FT_3_ levels, was independently associated with poor outcome and death [38]. In the aforementioned study, euthyroid sick syndrome was the most frequent thyroid disorder in a population of patients with mild COVID-19 infection and was an index of adverse outcome [38]. In a study performed in Italy, Baldelli et al. [39] evaluated thyroid hormone levels in COVID-19 patients, a group with pneumonia, a group with respiratory distress syndrome, and a group of controls and found significantly decreased FT_3_ and TSH levels in the patient groups, with lower levels found in the respiratory distress group. In a large retrospective study performed in Kroatia in severe COVID-19 patients, low TSH was observed. Sciacchitano et al. [40] studied patients with COVID-19 infection and hematological malignancies during the initial episodes of the SARS-CoV-2 pandemic in Rome, Italy, and found that low FT_3_ levels were related with increased neutrophil count, reduced T lymphocyte subpopulations, and augmented inflammation, tissue damage, and coagulation status. In the current study, low FT_3_ and TSH levels were observed in patients with severe COVID-19 infection. We found that euthyroid sick syndrome in our group of patients was significantly correlated with adverse outcomes, i.e., non-survival or death. In accordance with our findings, Iervasi et al. [41] noted that euthyroid sick syndrome is a strong predictor of death in patients with heart disease. Cerillo et al. [42] also found that low T_3_ syndrome may be a strong predictor of death in patients undergoing coronary artery surgery. We found that, in our group of severe SARS-CoV-2 disease patients, the presence of euthyroid sick syndrome was significantly related to disease severity and adverse outcome.

The pathophysiology of euthyroid sick or non-thyroidal illness syndrome is multifactorial and multifaceted. The thyroid hormone axis is centrally downregulated and induces a decrease in thyroid hormone secretion [43]. Euthyroid sick syndrome may be a manifestation of the acute phase response. Various inflammatory cytokines released during inflammation may alter thyroid secretion and hormone metabolism [44]. In particular, increased circulating interleukin-6 [45] and TNF-α [46], released during the acute phase response, reduce T_4_ metabolism to T_3_ leading to the euthyroid sick profile. In SARS-CoV-2 infection, an inflammatory cytokine release syndrome, described as “cytokine storm”, may be observed, which may be related to disease severity and even death [47,48]. The term cytokine storm describes the release of inflammatory cytokines and other inflammatory mediators during an infectious disease and is related to rapid disease progression and mortality [49]. The term was initially applied to describe graft versus host disease during allogeneic stem cell transplantation [47]. Subsequently, the term was further elucidated and applied in various disease states as well as in sepsis [50]. The role of the immune system is to recognize a foreign intruder in the organism and produce an inflammatory response in order to eliminate the foreign and possibly harmful agent. It also supports the repair of any damage induced by the foreign agent and the organism’s return to its initial basal condition. This immune system response is enhanced by cytokines, which are the means of communication between the immune cells as well as in the coordination of various immune cells. There appears to be a complex system of regulatory processes which maintain an equlibrium between inflammatory and anti-inflammatory cytokines and thus keep the immune response limited and balanced. If this balanced response of various mechanisms fails in one or more ways, it may induce excessive immune activation and augmented cytokine production, causing an unlocalized and systemic inflammatory response which may harm the organism [50]. The cytokine storm is shown to be characteristic of serious COVID-19 infection and characterized by augmented secretion of inflammatory cytokines, and it is related to disease severity [51]. The cytokine storm in COVID-19 disease is related to increased IL-6 and TNF-α expression [52]. IL-6 is implicated in the pathogenesis of euthyroid sick syndrome [53]. In acute myocardial infarction, low FT_3_, normal or low TSH, and high IL-6 and its soluble receptor were observed. IL-6 elevation and euthyroid sick syndrome development are interconnected [53]. Various data show a role of IL-6 in the interaction between thyroid axis and inflammatory mediators [17]. In a clinical study, IL-6 levels were negatively correlated with FT3 and positively with rT_3_ [43,54]. It may thus be that, in the case of acute and serious SARS-CoV-2 disease, the development of non-thyroidal illness syndrome may be linked to augmented inflammatory cytokine secretion [55].

In our study, we observed a negative correlation between CRP and FT_3_ levels, which was statistically significant, but the correlation was not statistically strong. CRP levels increase in acute infectious disease and are observed in acute COVID-19 infection [56]. CRP levels characterize acute SARS-CoV-2 infection. CRP synthesis by the liver is induced by IL-6. Elevated CRP levels in acute SARS-CoV-2 infection are related to the cytokine storm and to disease severity [56,57]. Thus, in our study, elevated CRP levels were found to be negatively related to FT_3_ levels.

Treatment of euthyroid sick syndrome remains a topic of high research interest [58]. Euthyroid sick syndrome may be considered an adaptive and energy preservation response [17]. Various investigators have attempted to treat patients with euthyroid sick syndrome with thyroxine, with controversial results. Thyroxine administration in the acute care unit as an intravenous infusion normalized T_4_ levels without affecting mortality [59]. Various studies have examined the effect of therapeutic T_3_ administration in adult patients with heart failure or coronary artery surgery. Other studies have shown promising as well as less promising results [60,61]. T_3_ administration for the preservation of organ function in brain-dead donors has been attempted in order to preserve functionality in the organs to be transplanted [62]. Combined treatment with GHRH, TRH, and GnRH in male patients with critical disease has also been attempted [63]. However, most researchers and clinicians agree that non-thyroidal illness syndrome should not be treated with thyroid drug administration [64], as the syndrome improves in parallel with the underlying illness. Euthyroid sick syndrome or non-thyroidal illness syndrome represents a response to fasting in healthy individuals and is present in patients with acute severe illness [65,66]. However, euthyroid sick syndrome in long-term critically ill patients, who have low T_3_, T_4_, and TSH levels, may represent a different facet of the disorder and may require treatment in order to prevent catabolic alterations within the organism [33]. It is this particular group of patients that may benefit from treatment for euthyroid sick syndrome and may be the subjects to benefit from new or developing treatments for the disorder [33,63].

Soon after the COVID-19 pandemic swept over the world, it became evident that the virus affected the thyroid gland, as numerous cases of subacute thyroiditis were described in relation with the infection [67,68,69,70]. Simultaneously, various cases of thyroiditis and autoimmune Hashimoto’s thyroiditis were described in association with SARS-CoV-2 infection. Additionally, new cases of Graves’ disease or exacerbation of previously stable Graves’ disease or Graves’ ophthalmopathy were described. Data emerged highlighting that the SARS-CoV-2 virus may be an autoimmune virus, i.e., a virus causing autoimmune diseases [71]. The COVID-19 virus invades the thyroid gland via the ACE2 enzyme, which the virus uses as a receptor and is expressed on thyroid cells. Prognosis of severe COVID-19 infection is a topic of interest in the current literature. Various indicators of severe disease and adverse outcome have been described, including vitamin D levels and kidney function parameters [72,73,74]. Herein, we describe the association of severe SARS-CoV-2 disease with euthyroid sick or non-thyroidal illness syndrome. The presence of euthyroid sick syndrome was related to disease severity and death. It appears that the measurement of thyroid hormone levels in patients admitted to hospital for COVID-19 disease may help to define disease severity and prognosis.

## 5. Study Limitations

This study has limitations as it utilizes a relatively small number of patients from a single center.

## 6. Conclusions

The SARS-CoV-2 virus led to a global pandemic and caused mild and severe disease. It also caused cases of severe pneumonia that could be lethal. The development of the cytokine storm, i.e., an inflammatory cascade, was related to severe disease and adverse outcomes. Early observations showed that the virus may affect the thyroid gland and induce subacute thyroiditis. Various cases of subacute thyroiditis have been described in the literature. It was later reported that the virus may also cause thyroiditis, autoimmune thyroiditis, hypothyroidism, and Graves’ disease or exacerbation of previously quiescent Graves’ disease or Graves’ ophthalmopathy. In the present study, we have provided evidence that patients hospitalized for severe SARS-CoV-2 disease may present with euthyroid sick syndrome or non-thyroidal illness syndrome or low T_3_ syndrome. The cytokine storm observed in severe SARS-CoV-2 disease may be related to the development of euthyroid sick syndrome. Data presented herein indicate that euthyroid sick syndrome in patients with severe SARS-CoV-2 disease may be related to disease severity and appears to be an indicator of adverse prognosis. The relationship between euthyroid sick syndrome and the cytokine storm may be further proof of the severe inflammatory reaction in severe COVID-19 infection. Thyroid hormone measurement in patients hospitalized for severe COVID-19 disease and the subsequent identification of the presence of euthyroid sick syndrome may be an index of disease severity and subsequent adverse outcomes.

## Figures and Tables

**Table 1:** Characteristics of patients admitted and cared for in hospital for severe COVID-19 infection: age (years) (mean ± SD), sex, white blood cell count, CRP (mg/L), ESR (mm/1 h), ferritin (ng/mL), d-dimers (μg/L), fibrinogen (ng/dL), K (mmol/l), PT (s), APTT (s), INR, and disease severity (1–4) [1—uncompromised respiratory function (pO2 > 70 mmHg, without need of oxygen supplementation); 2—mild respiratory insufficiency (pO2 50–60 mmHg, in need of oxygen supplementation with nasal cannula); 3—severe respiratory insufficiency (pO2 < 50 mmHg, in need of oxygen supplementation with high flow oxygen); 4—and severe respiratory insufficiency requiring intubation (pO2 < 60 mmHg on high flow oxygen supplementation)].

	SARS-CoV-2 Patients
Age	72.1 ± 16.09
Sex	33 F/30 M
White blood cell count (cells/μL)	6474.23 ± 3499.10
Neutrophils (%)	70.8 ± 13.4
CRP	65.1 ± 60.1
ESR (mm/1 h)	43.1 ± 23.9
Ferritin (ng/mL)	846.8 ± 125.1
d-dimers (μg/L)	1640.1 ± 1104.1
Fibrinogen (ng/dL)	547.5 ± 164.1
K (mmol/L)	3.87 ± 0.59
PT (s)	11.7 ± 1.05
APTT (s)	30.81 ± 3.96
INR	1.02 ± 0.1
Disease severity	1 (23), 2 (18), 3 (16), 4 (6)

## Data Availability

Data are available at the data archives of Asclepeion Hospital, Voula, Athens, Greece.
